# Glycation of paraoxonase-1 inhibits its activity and impairs the ability of high-density lipoprotein to metabolize membrane lipid hydroperoxides

**DOI:** 10.1111/j.1464-5491.2008.02546.x

**Published:** 2008-09

**Authors:** M Mastorikou, B Mackness, Y Liu, M Mackness

**Affiliations:** Division of Cardiovascular Sciences, University of Manchester, Department of MedicineManchester Royal Infirmary, Manchester, UK

**Keywords:** coronary heart disease, glycation, lipid hydroperoxides, paraoxonase-1, Type 2 diabetes

## Abstract

**Aims:**

High-density lipoprotein (HDL) protects against atherosclerosis development. Defective functioning of HDL in Type 2 diabetes may be one cause of increased cardiovascular disease associated with Type 2 diabetes. HDL modulates low-density lipoprotein and cell membrane oxidation through the action of paraoxonase-1 (PON1), which is one of the major mechanisms by which HDL is anti-atherogenic.

**Methods:**

We have compared the ability of HDL from Type 2 diabetic patients without coronary heart disease (CHD) (*n* = 36) to metabolize membrane lipid hydroperoxides with HDL from healthy control subjects (*n* = 19) and people with CHD but no diabetes (*n* = 37).

**Results:**

HDL from subjects with Type 2 diabetes and CHD metabolized 20% less membrane hydroperoxides than HDL from control subjects (*P* < 0.05). The PON1-192RR was least efficient in all the study groups. PON1 was glycated *in vivo*: (7.5% control, 12% CHD, 17% Type 2 diabetes *P* < 0.01) with QQ isoforms most glycated. *In vitro* glycation of PON1 reduced its ability to metabolize membrane hydroperoxides by 50% (*P* < 0.001); however, glyoxidation reduced it by 80% (*P* < 0.001). In the control group only there was a significant negative correlation between PON1 activity and the ability of HDL to metabolize membrane hydroperoxides (*r* = −0.911, *P* < 0.001).

**Conclusions:**

HDL from Type 2 diabetic patients without CHD has decreased ability to metabolize membrane lipid hydroperoxides, which could lead to increased susceptibility to cardiovascular disease.

## Introduction

The oxidation of low-density lipoprotein (LDL) in the arterial wall is believed to be the primary event leading to the initiation and progression of atherosclerosis [1,2]. High-density lipoprotein (HDL), however, is protective against the development of atherosclerosis [3,4]. The primary protective effect of HDL is believed to be its pivotal role in reverse-cholesterol transport; however, HDL also has anti-oxidative, anti-inflammatory and anti-thrombotic properties [5].

HDL-associated paraoxonase-1 (PON1) is primarily responsible for the anti-oxidative properties of HDL in retarding the oxidation of LDL and cell membranes [6–10]. By modulating the oxidation of LDL, PON1 abolishes the oxidized LDL stimulated induction of monocyte-chemotactic protein-1 (MCP-1) production by endothelial cells, thereby preventing monocyte/endothelial cell interaction in one of the earliest processes of atherosclerosis [11,12]. PON1 is low in subjects with Type 1 or Type 2 diabetes [13–15], leading to dysfunctional HDL with impaired antioxidant capacity [15]. In Type 2 diabetes there is an inverse relationship between PON1 activity and circulating oxidized LDL levels [16,17], indicative of the major role of PON1 in retarding LDL oxidation. We have recently shown that adenovirus mediated overexpression of human PON1 in a mouse model of metabolic syndrome significantly inhibits atherosclerosis development by reducing ox-LDL both in plasma and the artery wall [18].

Type 2 diabetes is associated with a 3- to 4-fold increased susceptibility in coronary heart disease (CHD) compared with people without diabetes and HDL cholesterol is lower than in non-diabetic subjects [19]. Increased glycation of HDL leads to the impairment of its anti-atherosclerotic properties [20]. *In vitro* glycation of HDL appears to partially inhibit PON1 [21]. We have previously shown that HDL from people with Type 2 diabetes but no coronary disease and HDL from people with coronary disease but no diabetes were defective in their capacity to metabolize oxidized-palmitoyl, arachidonyl phosphatidylcholine (ox-PAPC) (a primary pro-inflammatory product of LDL oxidation and PON1 substrate) compared with HDL from control subjects [22]. Previous studies indicated that HDL from people with Type 1 diabetes was significantly less efficient at metabolizing erythrocyte membrane hydroperoxides than HDL from normal control subjects [10]. It is therefore conceivable that HDL from Type 2 diabetic subjects will be defective in metabolizing cell membrane oxidized lipids and that this could contribute to increased atherosclerosis in Type 2 diabetes.

Previously, using these same populations, we showed that HDL from people with Type 2 diabetes or with CHD was significantly less able to metabolize ox-PAPC than HDL from control subjects [22], but we did not investigate the mechanism(s) responsible. In the present study, we show that this is also the case with the metabolism of cell membrane hydroperoxides. We also report that the likely mechanism behind this effect is the increased glycation of PON1 in people with Type 2 diabetes and those with CHD, which inhibits its activity.

## Patients and methods

### Study subjects

The study populations comprised 19 healthy control subjects attending for a routine health check matched for age (± 5 years) and gender with the patient groups. Thirty-six subjects with Type 2 diabetes were recruited from Manchester Royal Infirmary. Diabetes mellitus was diagnosed by World Health Organization (WHO) criteria [23]. All patients were receiving statin treatment, four were receiving calcium channel blockers, two angiotensin-converting enzyme (ACE) inhibitors, 20 metformin and two insulin, and none had CHD. Sixteen individuals were free of diabetic complications and 20 had one or more complication (16 with peripheral neuropathy, 10 with nephropathy and 11 with retinopathy). The third study group comprised 37 patients with angiographically assessed CHD and no diabetes, with > 70% stenosis of at least one coronary artery. All patients were receiving statins, seven were receivinh calcium channel blockers and five an ACE inhibitor. The Central Manchester Local Research Ethics Committee approved the study and all participants gave informed consent.

Venous blood was obtained after an overnight fast. Serum and EDTA plasma were obtained by low-speed centrifugation. Plasma and serum were stored at −20°C until analysis or used immediately to prepare HDL. DNA was extracted from lymphocytes.

### Biochemical analysis

Serum total cholesterol, triglycerides, HDL cholesterol, apolipoprotein A1 (apoA1) and B (apoB) were determined using a Cobas Bios II autoanalyser, using reagents and standards provided by the manufacturer (ABX Diagnostics, Shefford, UK). Glycated haemoglobin (HbA_1c_) was determined by HPLC using the Variant analyser (Bio-Rad Ltd, Hemel Hempstead, UK).

Serum PON1 activity towards paraoxon, PON1 concentration and PON1 genotypes (Q192R, L55M, C-108T) were determined by our previously published methods [13].

### Isolation of HDL

HDL (*d*1.063–1.21 g/l) was isolated by sequential ultracentrifugation as described [24].

### Preparation and oxidation of erythrocyte membranes

Human erythrocyte ghosts were prepared as described [10]. Briefly, heparinized blood samples of healthy subjects were collected after an overnight fast and centrifuged at 1468 *g*, 4°C for 20 min to separate the cells. Cells were then washed with 0.9% sodium chloride (NaCl) solution and incubated in 5 mm ice-cold phosphate-buffered saline (PBS) for 10 min to lyse the cells. The cells were centrifuged at 5000 *g* for 20 min to isolate the membranes, which were washed in saline until all haemoglobin was removed. Membrane protein concentration was determined by the bicinchoninic acid (BCA) method.

Erythrocyte membranes (5 mg) were oxidized by incubation with 5 mm 2,2-azo-bis(2-aminidinopropane) dihydrochloride (AAPH) for 20 h at 37°C. Oxidation was terminated by refrigeration and the membranes separated by centrifugation at 5000 *g* for 20 min. The oxidized membranes were re-suspended in saline. Oxidized erythrocyte membranes (500 µg) were incubated in the presence or absence of 100 µg HDL total protein, isolated from the study populations or glycated or glyoxidized HDL for 3 h at 37°C, after which time membranes were separated by centrifugation at 5000 *g* for 20 min and the membranes re-suspended in saline. Lipid hydroperoxides in the membranes and HDL were determined using the xylenol orange method [10].

### Glycation/glyoxidation of HDL

To prepare glycated HDL, HDL (1 mg) isolated from a pool of healthy control subjects was incubated in a final volume of 1 ml of N_2_-saturated PBS containing 40 mM BHT (butylated hydoxytoluene) and 25 mm glucose for 1 week at 37°C. Glyoxidation was achieved by incubating HDL with the omission of BHT and N_2_. Control incubations omitted glucose.

### Measurement of glycated PON1

The concentration of glycated PON1 in serum and HDL was determined by boronate affinity chromatography [25]. Briefly, serum or HDL were diluted 1 : 20 with PBS containing 1% triton X100 to dissociate the HDL complex and subject to m-aminophenylboronate affinity chromatography. Glycated and non-glycated PON1 were then determined by our in-house ELISA [13]. Briefly, standards, glycated and non-glycated PON1 samples were diluted 1 : 4500 in 0.05 m carbonate buffer pH 9.6, 100 µl added to duplicate wells of a 96-well plate and incubated for 16 h at room temperature (22°C). Wells were washed with PBS pH 7.4 containing 0.1% bovine serum albumin (PBS/BSA) and incubated with PBS/1% BSA for 1 h at room temperature. Following washing (× 3) rabbit anti-human PON1 IgG diluted 1 : 6400 in PBS/1% BSA was added and incubated for 1 h at room temperature. Wells were washed (× 2), anti-rabbit peroxidase conjugate (1 : 2500) added and incubated for 1 h at room temperature. Wells were then washed (× 3) and tetramethylbenzidine substrate added. After 15 min at room temperature, 2 msulphuric acid was added and the absorbance read at 450 nm. Recovery of PON1 in the two fractions was 96 ± 1.8% by reference to the original serum value.

### Statistical analysis

Statistical analysis was performed using SPSS 10.0 (SPSS Inc., Chicago, IL, USA). All parameters were tested for normality using the one-Sample Kolmogorov–Smirnov test. Statistically significant differences between variables with a Gaussian distribution were sought by Student's unpaired *t*-test. Variables with a non-Gaussian distribution were compared using the Mann–Whitney *U*-test. One-way anovawas used to show any effect of the PON1 polymorphisms on the variables investigated. Correlations between parameters were examined using Pearsons’ coefficient. The expected frequency of PON1 alleles were analysed by the Hardy–Weinberg equilibrium test. The Χ^2^-test was used to determine the significance of differences in allele frequency. All statistical analyses were conducted using SPSS version 10.0.

## Results

The demographic and clinical details of the study groups have been published previously [22] (Table 1). Compared with the control subjects, both the groups with Type 2 diabetes and CHD had higher serum triglycerides and significantly lower PON1 activity and concentration. The group with Type 2 diabetes had higher body mass index (BMI), while the group with CHD had lower HDL cholesterol and apoA1. Serum total cholesterol was not significantly different between the groups, probably because all the patients were receiving statin therapy. There were no differences in the distribution of the PON1-L55M, Q192R or C-108T polymorphisms between the groups [22].

**Table 1:** Characteristics of the study groups

Parameters	Control	Type 2 diabetes	CHD
*n* (male)	19 (10)	36 (21)	37 (22)
Age (years)	57.7 ± 4.8	57.7 ± 5.2	57.7 ± 5.4
BMI (kg/m^2^)	21.2 ± 3.2	28.8 ± 5.0**	25.3 ± 4.0
HbA_1c_ (%)	—	7.6 ± 1.4	—
Total cholesterol (mmol/l)	4.8 ± 1.6	5.1 ± 1.5	4.4 ± 1.0
Triglycerides (mmol/l)†	1.05 (0.51–3.27)	2.24** (0.68–9.27)	1.73** (0.64–3.21)
HDL cholesterol (mmol/l)	1.55 ± 0.41	1.29 ± 0.59	1.05 ± 0.34**
ApoB (mg/dl)	78.0 ± 59.5	88.0 ± 31.2	82.3 ± 21.0
ApoA1 (mg/dl)	110.5 ± 38.3	128.7 ± 46.3	85.1 ± 20.0**
PON1 activity† (nmol/min/ml)	269.4 (68.2–487.2)	113.6** (40.2–409.4)	150.9** (40.2–463.8)
PON1 mass† (µg/ml)	113.1 (35.2–239)	90.1* (17.6–175.9)	83.5* (19.4–124.9)

Significantly different from control

* *P* < 0.05,

** *P* < 0.01.

Values are mean ± sd except

† which are median (range).

Apo, apolipoprotein; BMI, body mass index; CHD, coronary heart disease; HbA_1c_, glycated haemoglobin; HDL, high-density lipoprotein; PON1, paraoxonase-1; sd, standard deviation.

**FIGURE 1:**
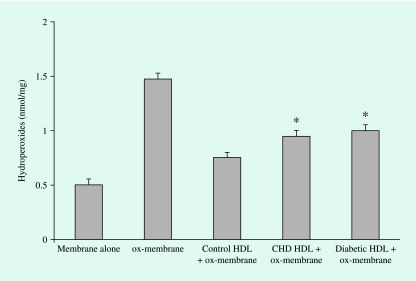
Metabolism of membrane lipid hydroperoxides by HDL from the three study populations. Oxidized erythrocyte membranes (500 µg) were incubated in the presence or absence of 100 µg HDL for 3 h at 37°C. Membranes were isolated and hydroperoxides measured as described in Patients and methods. Data are mean ± sd. Significantly different from HDL from control subjects. *P < 0.05. CHD, coronary heart disease; HDL, high-density lipoprotein; ox, oxidized; sd, standard deviation.

**FIGURE 2:**
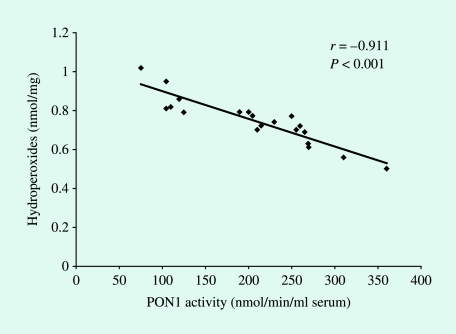
Effect of paraoxonase-1 (PON1) activity on the ability of high-density lipoprotein (HDL) to metabolize membrane hydroperoxides. Experimental details can be found in Patients and methods.

HDL from the three study populations was able to metabolize cell membrane lipid hydroperoxides (Fig. 1); however, HDL from people with Type 2 diabetes and CHD were significantly less able to metabolize membrane hydroperoxides than HDL from control subjects (both *P* < 0.05), by 20–25%. The concentration of lipid peroxides in the HDL fractions did not differ between the groups (result not shown) nor was it higher than in control incubations with unoxidized membranes, indicating metabolism of the membrane hydroperoxides rather than simple transfer to HDL. In the control group, there was a significant negative correlation (*r* = −0.911, *P* < 0.001) between the ability of HDL to metabolize membrane hydroperoxides and PON1 activity (Fig. 2), indicating a primary role for PON1 in the metabolism of these hydroperoxides. Although there were correlations between PON1 activity and HDL hydroperoxide metabolism, these did not reach statistical significance in the Type 2 diabetes (*r* = −0.624, *P* = 0.058) or CHD (*r* = −0.598, *P* = 0.06) groups.

**FIGURE 3:**
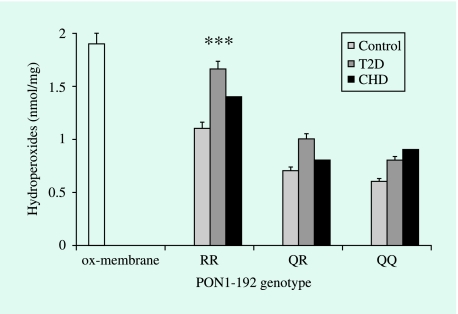
Effect of the PON1-192 genotype on the ability of HDL to metabolize membrane hydroperoxides. PON1 genotypes were determined by PCR/RFLP analysis. Membrane hydroperoxides were determined as described in Fig. 1. Data are mean ± sd. Significantly different from the PON1-192 QQ and QR genotypes *P < 0.01. CHD, coronary heart disease; HDL, high-density lipoprotein; PON1, paraoxonase-1; sd, standard deviation; T2D, Type 2 diabetes.

In all three study groups, HDL containing the PON1-192RR isoform was significantly (*P* < 0.01) less able to metabolize membrane hydroperoxides than HDL containing the PON1-192Q isoform (Fig. 3). There was no effect of the PON1-55 or PON1-108 polymorphisms.

**FIGURE 4:**
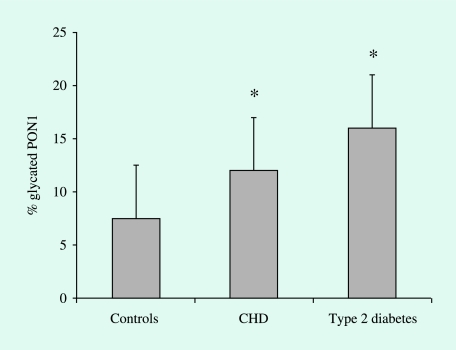
Percentage of glycated paraoxonase-1 (PON1) in sera from control, Type 2 diabetic patients and patients with coronary heart disease (CHD). Serum glycated and non-glycated PON1 were separated by boronate affinity chromatography and their concentrations measured by ELISA as described in Patients and methods. Data are mean ± sd(standard deviation). Significantly different from control subjects *P < 0.01.

**FIGURE 5:**
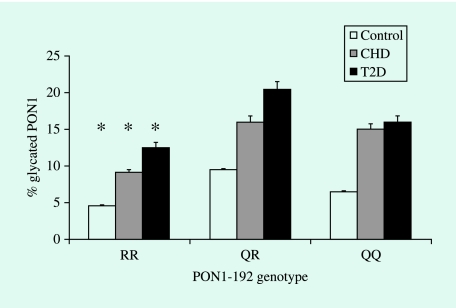
Percentage of glycation of PON1 isoforms according to PON1-192 genotype. See Figs 3 and 4 for experimental details. Data are mean ± sd. Significantly different from the PON1-192 QQ and QR genotypes *P < 0.001. CHD, coronary heart disease; PON1, paraoxonase-1; sd, standard deviation; T2D, Type 2 diabetes.

The amount of glycated PON1 in serum increased from 7.5% in control subjects to 12% in those with CHD and 17% in those with Type 2 diabetes (both *P* < 0.01) (Fig. 4). The PON1-192 genotype, but not the PON1-55 or PON1-108 genotypes, affected the amount of glycation of PON1 (Fig. 5). In all three study populations, the RR isoform was significantly less glycated (*P* < 0.01) than either the QR and QQ isoforms, indicating possible structural differences between the PON1 isoforms.

**FIGURE 6:**
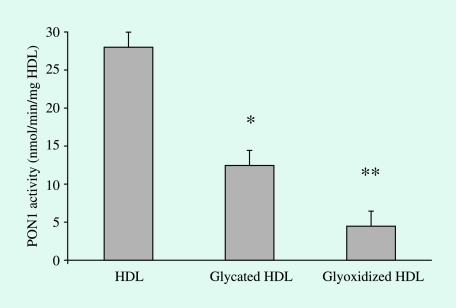
Effect of glycation/glyoxidation on paraoxonase-1 (PON1) activity towards paraoxon. PON1 activity was determined using paraoxon as substrate in 100 µg control high-density lipoprotein (HDL) or HDL glycated or glyoxidized as described in the Patients and methods. Activity data for control HDL is presented after 7 days’ incubation. Compared with HDL before incubation the activity loss is 10 ± 0.9%. Data are mean ± sd(standard deviation). Significantly different from control HDL *P < 0.005, **P < 0.001.

**FIGURE 7:**
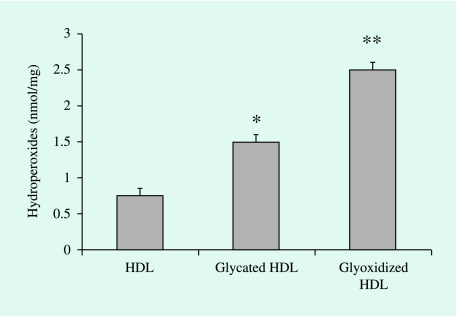
Effect of glycation/glyoxidation on paraoxonase-1 (PON1) activity towards membrane hydroperoxides. One-hundred-microgram quantities of control, glycated or glyoxidized high-density lipoprotein (HDL) were incubated with oxidized erythrocyte membranes and hydroperoxides determined as described in Fig. 1. Data are mean ± sd (standard deviation). Significantly different from control HDL *P < 0.01, **P < 0.001.

We next investigated whether the glycation and glyoxidation of PON1 on HDL was a reason for the perturbed ability of HDL to metabolize membrane hydroperoxides. PON1 activity towards paraoxon was reduced by 60% in glycated HDL and by over 80% in glyoxidized HDL (*P* < 0.001) compared with normal HDL (Fig. 6). Similarly, compared with normal HDL, the ability of glycated HDL to metabolize membrane hydroperoxides was reduced by 50% (*P* < 0.001) and that of glyoxidized HDL by 80% (*P* < 0.001) (Fig. 7).

## Discussion

We have shown for the first time that a significant fraction of PON1 is glycated *in vivo* and that approximately twice as much PON1 from people with Type 2 diabetes and those with CHD is glycated compared with control subjects. Although none of our CHD population had elevated glucose at the time of study, we cannot exclude previous transient hyperglycaemic episodes, for example, postprandially, as a reason for the increased glycation of PON1, nor can we exclude the possibility of increased PON1 half-life in this population. Interestingly, in a previous study, apoB was found to be glycated in the order of control < CHD < Type 2 diabetes, with no difference in fasting glucose between control subjects and those with CHD [25]. We have also shown that HDL from people with Type 2 diabetes and those with CHD is significantly less able to metabolize erythrocyte membrane lipid hydroperoxides than control HDL and there is a significant linear relationship between lipid hydroperoxide metabolism and HDL-PON1 activity in control subjects but not in people with Type 2 diabetes or those with CHD, indicating a derangement in the ability of HDL to metabolize lipid peroxides in these people as we have found previously [22]. Very similar results were reported previously for HDL from people with Type 1 diabetes [10].

It has previously been reported that *in vitro* glycation inhibits PON1 activity towards paraoxon [21]. In this study we found that glycation of PON1 inhibited both paraoxon hydrolysis and membrane lipid hydroperoxide metabolism by approximately 50%; however, glyoxidation caused 80% inhibition of these two PON1 activities. It is therefore possible that the increased *in vivo* glycation of PON1 leads to its glyoxidation and is responsible for the derangement of membrane hydroperoxide metabolism found in HDL from people with Type 2 diabetes and those with CHD. Modification of HDL by glycation or oxidation affects its anti-atherosclerotic properties such as reverse-cholesterol transport [20]. Having deranged antioxidant activity towards membrane hydroperoxides would lead to increased oxidation of cells in the artery wall, increased inflammation in the vessel wall and increased atherosclerosis [26]. Although inhibition of PON1 by glycation appears to be the most likely cause of our results, we cannot discount the possibility that glycation of other HDL proteins such as apoA1 or lecithin : cholesterol acyltransferase effects PON1 activity and/or the ability of HDL to metabolize hydroperoxides. This could be by disrupting HDL structure, or glycation of apoA1 could effect its ability to modulate PON1 function.

There were significant differences in both activity towards membrane hydroperoxides and glycation of PON1 in the PON1-192 but not the PON1-55 or PON1-108 genotypes. The PON1-192R isoform was least efficient at metabolizing membrane hydroperoxides, but also the least glycated. At first this may seem to contradict the results presented earlier. However, there are major structural differences between the Q and R isoforms of PON1 [27]. The amino acid at PON1 position 192 is involved in HDL binding. The PON1-192Q isoform binds to HDL with threefold lower affinity than the R isoform and as a consequence has reduced stability, activity and ability to promote macrophage cholesterol efflux [27]. It is possible that the additional stability of the R isoform allows it to resist inhibition by glycation, whereas the relatively unstable Q isoform cannot.

Rosenblat *et al*. [28] have shown that some serum PON1 is present in the lipoprotein deficient fraction (LPDS) and that this enzyme is less able to reduce LDL oxidation or promote macrophage cholesterol efflux than HDL-associated PON1. These authors also found that there was significantly more PON1 present in LPDS from subjects with Type 2 diabetes than healthy control subjects and that this may be one reason for the accelerated atherosclerosis associated with Type 2 diabetes. However, this is unlikely to affect the results presented here, as we used only isolated HDL, and it is more likely that a direct inhibition of PON1 by glycation/glyoxidation occurred. These results may also help to explain why PON1 is an independent risk factor for coronary events [29].

The PON1-Q192R polymorphism determines a substrate-dependent polymorphism [30]. Whereas some, such as paraoxon, are hydrolysed more efficiently by the R isoform, others are hydrolysed more efficiently by the Q isoform [31]. We and others have shown that the R isoform is least efficient at metabolizing a number of oxidized lipids, such as oxidized PAPC [22,32,33]. In our current study, the R isoform was least able to metabolize membrane hydroperoxides, consistent with previous findings.

In conclusion, in people with Type 2 diabetes and those with CHD, PON1 is heavily glycated *in vivo*. This appears to inhibit its ability to metabolize membrane lipid hydroperoxides. Although the differences between the populations are small, they are significant and could contribute to greater inflammation in the vessel wall and increased atherosclerosis in these populations.

## Competing interests

Nothing to declare.

## Abbreviations

ACE: angiotensin-converting enzyme
CHD: coronary heart disease
HDL: high-density lipoprotein
LDL: low-density lipoprotein
ox: oxidized
PAPC: palmitoyl, arachidonyl phosphatidylcholine
PBS: phosphate-buffered saline
PON1: paraoxonase-1
